# Characterizing Dynamic Changes in the Human Blood Transcriptional Network

**DOI:** 10.1371/journal.pcbi.1000671

**Published:** 2010-02-12

**Authors:** Jun Zhu, Yanqing Chen, Amy S. Leonardson, Kai Wang, John R. Lamb, Valur Emilsson, Eric E. Schadt

**Affiliations:** 1 Department of Genetics, Rosetta Inpharmatics, LLC, a wholly owned subsidiary of Merck & Co., Inc., Seattle, Washington, United States of America; 2 Molecular Profiling and Research Informatics Department, Merck Research Laboratories, Rahway, New Jersey, United States of America; University of Tokyo, Japan

## Abstract

Gene expression data generated systematically in a given system over multiple time points provides a source of perturbation that can be leveraged to infer causal relationships among genes explaining network changes. Previously, we showed that food intake has a large impact on blood gene expression patterns and that these responses, either in terms of gene expression level or gene-gene connectivity, are strongly associated with metabolic diseases. In this study, we explored which genes drive the changes of gene expression patterns in response to time and food intake. We applied the Granger causality test and the dynamic Bayesian network to gene expression data generated from blood samples collected at multiple time points during the course of a day. The simulation result shows that combining many short time series together is as powerful to infer Granger causality as using a single long time series. Using the Granger causality test, we identified genes that were supported as the most likely causal candidates for the coordinated temporal changes in the network. These results show that *PER1* is a key regulator of the blood transcriptional network, in which multiple biological processes are under circadian rhythm regulation. The fasted and fed dynamic Bayesian networks showed that over 72% of dynamic connections are self links. Finally, we show that different processes such as inflammation and lipid metabolism, which are disconnected in the static network, become dynamically linked in response to food intake, which would suggest that increasing nutritional load leads to coordinate regulation of these biological processes. In conclusion, our results suggest that food intake has a profound impact on the dynamic co-regulation of multiple biological processes, such as metabolism, immune response, apoptosis and circadian rhythm. The results could have broader implications for the design of studies of disease association and drug response in clinical trials.

## Author Summary

Peripheral blood is the most readily accessible human tissue for clinical studies and experimental research more generally. Large-scale molecular profiling technologies have enabled measurements of mRNA expression on the scale of whole genomes. Understanding the relationships between human blood gene expression profiles and clinical traits is extremely useful for inferring causal factors for human disease and for studying drug response. Biological pathways and the complex behaviors they induce are not static, but change dynamically in response to external factors such as intake/uptake of nutrients and administration of drugs. We employed a randomized, two-arm cross-over design to assess the effects of fasting and feeding on the dynamic changes of blood transcriptional network. Our work has convincingly shown that feeding or increasing nutritional load affects the human circadian rhythm system which connects to other biological processes including metabolic and immune responses. We believe this is a first step towards a more comprehensive population-based study that seeks to connect changes in the blood transcriptome to drug response, and to disease and biology more generally.

## Introduction

Elucidating networks that define biological pathways underlying complex biological processes is an important goal of systems biology. Large-scale molecular profiling technologies have enabled measurements of mRNA and protein expression on the scale of whole genomes. As a result, understanding the relationships between genes and clinical traits, and inferring gene networks that better define biochemical pathways that drive biological processes, has become a major challenge to understanding large-scale data sets generated from these technologies. For the majority of published gene expression profiling experiments, they are carried out at a single pre-defined time point across all samples, where the implicit assumption is that the steady state for the corresponding biological system is well approximated at a single time point. The steady state in this context represents a baseline state of the system under study in which the system is least likely to change and has the least amount of variability due to environment.

Because biological pathways and the complex behaviors they induce are dynamic [1], transcriptional response, interactions among proteins and other such processes, take time and ultimately lead to time-dependent variations in mRNA, protein and metabolite levels. These types of temporal variation over time are difficult to study directly with measurements taken at only a single time point. Recently, studies applying time series to temporal gene expression data have been published, covering a range of experiments focusing for instance on the SOS DNA repair system in *E.coli* [2], the cell cycle in yeast [3], muscle development in *Drosophila* [4] and cell cycle processes in human cell lines [5]–[6].

Coexpression networks are based on pair-wise gene-gene correlations of expression data, revealing functional modules in the network that elucidate pathways that drive core biological processes [7]–[8] or pathways that underlie complex human disease [9]–[10]. Coexpression networks provide global views of network structures, but by themselves cannot yield causal relationship between genes or between genes and clinical traits. Using a Bayesian network approach to integrate genetic, expression, and clinical data in segregating populations, we have previously demonstrated that such causal relationships can be inferred [11]–[14]. While these network approaches have proven useful in elucidating complex traits emerging in complex systems at the population level, they have however been based on data sampled at a single time point.

**Figure 1:**
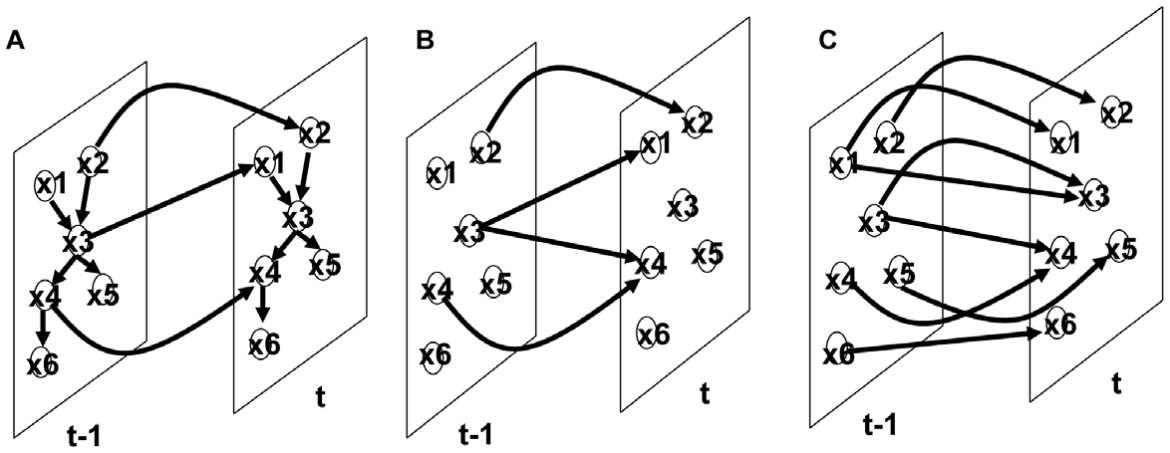
Dynamic Bayesian models under different assumptions. (A) a general two-slice model: a DBN under first-order Markov assumption; (B) a simplified two-slice model assuming no intra-slice interactions; (C) a two-slice model assuming that every variable is under self regulation.

A static Bayesian network (SBN) is a graphical model that encodes a joint probability distribution 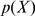 on a set of stochastic variables 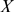, which can be decomposed as 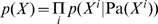, where 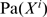 represents the parent set of 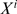. Similar to a static Bayesian network, a dynamic Bayesian Network (DBN) is also a graphical model with a joint probability distribution. The main difference between them is that DBN also captures temporal relationships between variables 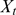 which is the vector for variables 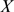 at the time point 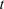. If there are 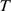 time points, then the joint probability distribution 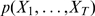 can be decomposed as 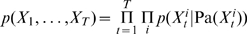, where 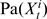 represents the parent set of 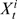. In general, 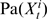 can include variables from the same time point 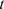 or the previous time points (represented as 
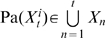
). There are many ways to simplify the complexity of the DBN model and data required to train the model. First, we can assume first order Markov property for transitional dependence, then the parent set can be simplified as 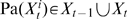 which corresponds to a general two-slice model (Figure 1A). The intra-slice links represent causal relationships inferred at static states or causal relationships happens in a shorter time than the sampling time between 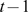 and 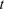. We will refer to this model as DBN in our present study. Second, we can further simplify the model and assume 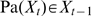 (the variables in current time 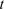 only depend on the previous time point 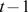), then the DBN corresponds to a simplified two-slice model without intra-slice interactions (Figure 1B). Third, if we assume that the variable 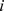 is self regulated (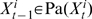), then the DBN can be represented as a two-slice model in Figure 1C, which is equivalent to a Granger causality test with a stationary Bivariate Auto-Regressive model (BVAR). We will refer this model as the Granger causality test in our result.

The DBN is a popular approach in computer sciences, such as Kalman filter and Hidden Markov Model (HMM) in voice recognition [15] or more recently in inferring transcriptional regulatory networks from time series data [2] and protein fragmentation process [16]. Another independent line of research of inferring causal relationship from time series is “Granger causality”. The Granger causality concept was originally developed for economic time series data [17], but has since been applied to time series data in many different domains. The Granger causality networks under some assumptions are similar to special cases of the DBN. For example, the model in Figure 1C is a DBN and a Granger causality network with a stationary BVAR model. However, while the Granger causality and the DBN have recently been applied to elucidate temporal causality networks in a number of experimental works, such as SOS DNA repair in *E.coli* [2], cell cycle in yeast [3], muscle development in *Drosophila* [4], and cell cycle in human cell lines [5]–[6], no studies to our knowledge have expanded on this concept of temporal causality to gene expression time series data collected *in vivo* in humans.

One of challenges of applying the Granger causality test to human samples is how to generate long time series data. We overcome the problem by combining multiple short time series. Our simulation results show that data combined from multiple short time series is as informative as a long time series. One of challenges of applying DBN to human samples is limited sample size. We tackled this problem by reconstructing the intra-slice structure from a large data set generated at static states, then reconstructing the inter-slice structure from the time series data.

**Figure 2:**
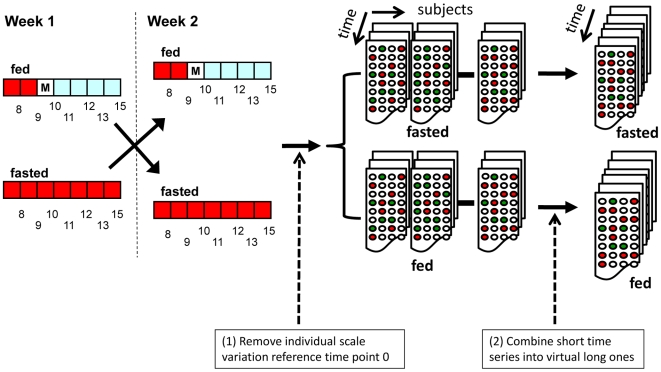
Experimental design and data processing scheme. Forty healthy volunteers were recruited to participate in the study and randomized to a treatment arm (either fasted or fed). To minimize individual scale differences, every participant's gene expression profile after the first time point was referenced (re-ratioed) to their corresponding expression profile at time point 0. The re-referenced gene expression values from time point 1 to 6 in the figure represent the expression data used herein as virtual long time series.

In the present study we have applied methods based on Granger causality and DBN to a set of human blood gene expression profiles generated at multiple time points during the course of a day, shown in Figure 2. The blood gene expression data was generated from 40 apparently healthy males participating in a randomized, two-arm cross-over design study to assess the effects of fasting and feeding on the blood transcriptional network [18] (see Materials and Methods section for details). The fasted and fed arms of the study provided the necessary data to characterize the dynamic changes in gene expression and corresponding pathways associated with fasting and feeding states in human blood samples [18]. After removing individual scaling effects by referencing individual's time point 0, short time series were combined into virtual long time series (shown in Figure 2). Using the Granger causality test, we identified *PER1* as the key regulator of the blood gene expression pattern in which multiple biological processes were under circadian rhythm regulation. Furthermore, the genes under *PER1* regulation in the fed network are enriched for obesity causal genes. Finally, using the DBN, we show that over 72% of all inter-slice links are self links and when the SBN and the DBN were compared, we found that different processes such as inflammation and lipid metabolism, which are disconnected during fasting, are now dynamically linked together in response to food intake.

## Results

### Identifying causal regulators using Granger causality

The two-way or three-way ANOVA analysis defining time- and state-dependent gene expression signatures provides meaningful way to characterize expression changes on a global scale [18]. However, these methods on their own do not provide any information on the causal regulators driving the time-dependent gene expression behavior. To leverage the time series data more maximally towards this end, we applied Granger causality test to gene expression traits scored systematically in the fasted/fed cohort blood samples at roughly 1 hour intervals during the course of a day (Figure 2). A gene expression trait 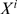 is said to be Granger causal for gene expression trait 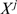 if, at previous time points, 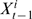 provides significantly more information on time-dependent changes in 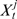 than the historical information 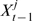 provides on itself. In our implementation of the Granger causality test, we test this by fitting 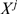 to an autoregressive model with respect to the different time points, and then testing whether extending the autoregressive model by including 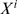 improves the fit (see Materials and Methods for details). If there is a statistically significant improvement testing the model fit (assessed by comparing the models using the F test), then we declare that 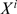 is Granger causal for 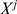, or simply as 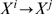.

Traditionally, a long time series is required to apply Granger causality test. However, it is hard to obtain a long time series of human samples collected *in vivo*. We have previously shown that over 80% of transcripts have significant inter-individual variances [18], which is comparable to previously reported result [19]. Thus, we can treat time series data from 40 patients as 40 independent short time series. Assuming these 40 time series have similar dynamic behavior, but with different starting points, we can combine them together to generate a virtual long time series (shown in Figure 2, and see Materials and Methods for details). Our simulation results show that the virtual long time series are as informative as long time series with similar data points (shown as Supplementary Figures S1 and S2). We constructed causal networks for the fasted and fed states by applying the Granger causality test to all gene expression trait pairs generated in the fasting/feeding cohort described in Figure 2. For gene expression traits 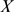 scored in the fasting/feeding cohort, a link 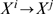 was inserted into the causal network if the p-value associated with the Granger causality test was less than 0.01 after multiple testing correction. The resulting fasted and fed networks were comprised of 2010 and 967 causal links (listed in Supplementary Tables S1 and S2), respectively. The corresponding false discovery rates (FDR) [20] for the causal links in the fasted and fed networks were 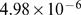 and 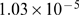, respectively. Bootstrapping test results (see Materials and Methods for details) show that 80% and 90% of links in fast and fed networks have confident values above 0.5, respectively (shown in Supplementary Figure S3). Both networks were observed to exhibit the scale-free property for out-degree distributions (shown as Supplementary Figure S4). From these data it was possible to identify all expression traits supported as Granger causal for at least one other expression trait in the network (referred to here as causal regulators), and then rank order the causal regulators according to the number of genes for which they were supported as causal, shown in Table 1.

**Table 1:** Top causal regulators in the fast and fed Granger causality networks.

Fasting			Fed		
Accession	gene	out-degree	Accession	gene	out-degree
AJ420555	RNF144B	53	NM_002616	PER1	211
AK057742	C10orf46	41	RSE_00000601195	SLC22A23	68
AF038535	SYT7	31	NM_003869	CES2	17
NM_006868	RAB31	31	NM_001657	AREG	16
NM_052868	IGSF8	27	NM_007246	KLHL2	8
NM_002483	CEACAM6	21	Contig53615_RC	Contig53615_RC	8
NM_024075	TSEN34	21	NM_001875	CPS1	8
NM_004090	DUSP3	20	NM_024321	RBM42	8
NM_021943	ZFAND3	20	NM_000967	RPL3	8

**Figure 3:**
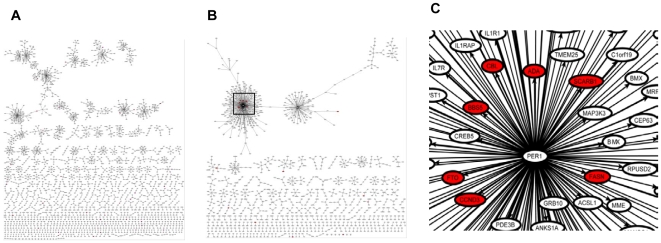
Fasted and fed Granger causality networks. (A) the global view of the fast network; (B) the global view of the fed network; (C) the zoom-in view of the subnetwork around *PER1* (black box) in (B). There are more links in the fast networks. The fast network consists of many small subnetworks and the fed network consists of two large subnetworks and a few small ones. Nodes in red are obesity causal genes.

There are more causal links inferred for fast time series than for fed time series. The fasted network consists of many small subnetworks and the fed network consists of mainly two subnetworks (shown in Figures 3A and 3B). The top causal gene in the fasted network is *RNF144B*, a putative ubiquitin-protein ligase that plays a role in mediating p53-dependent apoptosis. Genes under *RNF144B* regulation including *PTEN* are enriched for the GO biological process of negative regulation of cellular metabolic process (p-value = 0.008). The top causal gene in the fed network is *PER1*, a transcription factor regulating the circadian clock, cell growth and apoptosis. The genes under *PER1* regulation are enriched for genes correlated to plasma concentration of triglyceride (p-value = 0.00045) in the Icelandic Family Blood (IFB) cohort [10]. *PER1*'s downstream genes are involved in diverse biological processes including *CREB5*, in circadian rhythm, *PTEN* and *P53INP2* in apoptosis, *IL1R1*, *IL1RAP* and *TLR2* all involved in inflammation response, *FASN* and *ACSL1* in fatty acid metabolism and *MVK* in cholesterol biosynthesis. These results suggest that food intake interacts with circadian rhythm and the circadian rhythm has impacts on many biological processes as has been previously shown in mouse studies [21]–[22]. Further, previous research has demonstrated circadian gene (*PER1*, *PER2*, *PER3* etc.) mRNA expression rhythm in human peripheral blood cells and linked that to individual's circadian phenotype [23]–[24]. Our blood causal network where *PER1* is a top causal gene illustrates a potential mechanism of how the CNS control and environmental influences (e.g. external sunlight) can affect circadian rhythm gene expression which in turn regulating a host of other biological functions. More specifically, circadian rhythm genes (*PER1* in particular) play important roles in cell cycle regulation and cancer processes [25]–[26]. These reports support our observations in the fed network that several genes under *PER1* control are involved in apoptosis and cell cycle regulation (e.g., *PTEN* and *P53INP2*).

380 human genes are cataloged as obesity causal genes in the human obesity map [27]. In recent years, many large genome-wide association studies (GWAS) have convincingly identified a number of genes causing human obesity. 34 genes including *FTO* were replicated in many populations [28]–[31] Taking consideration of these two sources, there are 409 obesity causal genes, and 246 of them were expressed in our blood data set. When the obesity causal genes were overlapped with the fasted and fed networks, 7 genes (*ADA*, *BBS5*, *CBL*, *CCND3*, *FASN*, *FTO* and *SCARB1*) overlapped with *PER1*'s downstream genes in the fed network (Fisher's Exact Test p-value = 0.037) (shown in Figure 3C). It has been shown that circadian rhythm links to metabolic processes in mouse [32]–[33]. For instance, mutations in mouse genes involving circadian rhythm regulation, such as *Clock*, can lead to obesity [34]. Our results provide evidence that human obesity causal genes are under circadian rhythm control in a peripheral tissue like blood.

### Connecting different biological processes using dynamic Bayesian networks (DBN)

Constructing DBN using the model described in Figure 1A, requires a large amount of data and computational resources. However, when the intra-slice structure (the SBN) is known, then there is a dramatically reduced demand for large amounts of both data and computational resources. A large dataset of profiled peripheral blood samples (IFB) is already described and available [10]. The fasting feeding study group and the IFB cohort are derived from the same population both in terms of geological location and genetic background, therefore the static networks based on these two studies are assumed to be similar. The IFB data set consists of both gene expression measured in the fasting state and genotype data. Previously, we demonstrated that Bayesian networks constructed by integrating gene expression data and genotype data were of high quality [12]–[13],[35]. To match for gender, data from 455 males in the IFB cohort was used to construct a static Bayesian network which consisted of 7310 nodes (genes) and 11047 links (see Method Section for details). The static Bayesian network was fixed as the intra-slice network in the DBN model shown in Figure 1A, and then the time series data (fast or fed) were used to construct inter-slice connections.

The fasted and fed DBNs consisted of 1125 and 1290 inter-slice links (listed in Supplementary Tables S3 and S4), respectively. Among them, 846 (75%) and 936 (73%) were self links. 404 self links are common between the fasted and fed DBNs. The genes under self control (with self links in DBNs) are enriched for *cis* expression quantitative traits (*cis* eQTLs) in blood (enrichment p-values = 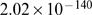 and 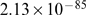 for the fasted and fed DBNs, respectively).

**Figure 4:**
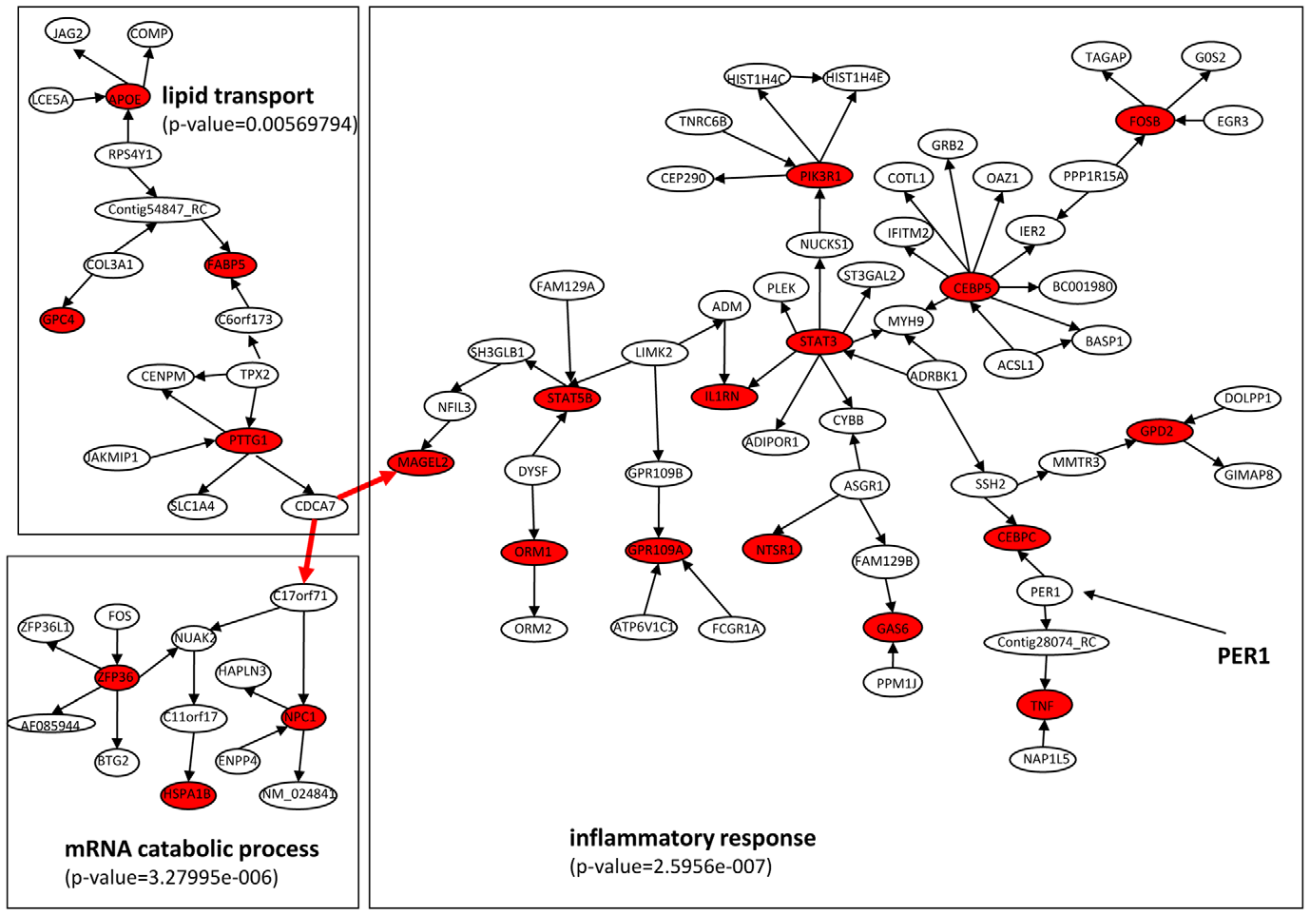
Changes in the obesity causal genes subnetworks between the fed DBN and the SBN. Nodes in red represent obesity causal genes. Edges in black are links in the SBN and Edge in red are inter-slice links in the fed DBN. Three subnetworks in the BN are connected in the fed DBN. The circadian rhythm regulator *PER1* is also in the subnetwork.

One important goal for utilizing time series data is to study the dynamic changes in molecular networks. Under static condition, many biological processes may be disconnected or loosely connected, whereas under a perturbation, these processes will change coordinately. 409 obesity causal genes mentioned above were collected from two resources, namely the human obesity map [27] and recent GWAS data [28]–[29],[36]. 138 out of the 409 genes are included in the DBNs. These 138 genes were used as seeds to construct obesity related sub-networks for fast and fed DBN and the SBN as previously described [13]. The fasted and fed subnetworks were compared with the subnetworks constructed from the SBN. The largest change was from the fed subnetwork, where three segmented subnetworks in the SBN were connected in the fed DBN by two inter-slice links (shown as red in Figure 4). *CDCA7*, a transcription regulator for the cell cycle, is found in the center of the connected subnetworks. It connects genes involved in lipid metabolism such as *NPC1*, *FABP5* and *APOE* to the large subnetwork on the left which consists of inflammatory response genes such as *STAT3*, *STAT5*, *GPR109A*, *TNF*, *NTSR1*, *ORM1* and *IL1RN*. This suggests that the expression of genes involved in either inflammatory response or lipid metabolism change coordinately in response to food intake. It is also worth noting that the circadian rhythm regulator *PER1* is in the subnetwork on the left, which consists of many genes involved in inflammatory response pathways. As well, in the fed DBN, both cell cycle regulation and lipid metabolism processes are linked to the circadian rhythm.

## Discussion

Designing experiments to generate large-scale molecular phenotyping data and to enable inferring causal relationships among genes and between genes and clinical endpoints is now a feasible task. Genetic variants (e.g. nonsynonimous, nonsense, eSNPs etc), genetically modified animals (e.g., knockouts, transgenics, RNAi knockdown), and chemical perturbations have all been used successfully to establish a causal relationship between genes and phenotypes in mammalian systems. Here we have detailed the use of time series data in a human population to predict causal regulators using a Granger causality test and a DBN. Our Granger causality networks showed that multiple biological processes such as apoptosis, inflammation response and lipid metabolism are under circadian rhythm regulation and obesity causal genes are under circadian rhythm regulator *PER1* in the fed networks. For the DBN, we showed that over 73% of inter-slice links are self links. When the SBN and the DBN were compared, we find that different processes such as inflammation and lipid metabolism are linked together during the dynamic changes in responding to food intake.

The time series data provided a path to go beyond the characterization of interesting patterns of expression and network differences associated with complex states (like fasting and feed status), by allowing for the identification of putative causal regulators driving these differences. While extensive experimental validation will be required to assess the full utility of the approach detailed in the present study, we believe these methods and the characterizations of time and state dependent changes in gene expression and network topology, will motivate a need to integrate a time domain into gene expression experiments that aim to elucidate complex system behavior.

Our data consist of many short time series from multiple individuals instead of a single long time series. Our approach for combining multiple short time series was based on the assumption that individual response slopes are similar. First, the population under study is relatively homogeneous, i.e. only males, similar age, same population, same ethnicity and each individual consumed the meal of same size and composition. Second, we reduced the individual specific variance by normalizing each individual data according to its own expression data at the first time point. This essentially reduces the number of parameters to fit in the model, at the cost of reducing the number of time points available to feed into the model. In contrast, if the population under study was genetically heterogeneous, we would treat the response slope differently for different individuals and would employ the mixed-effects model as suggested by Berhane and Thomas [37] for combining time series. In that case, we wouldn't need to normalize data for each individual, and as a result there would be an increase in the number of parameters to fit as well as an increase in the available data points. We note in passing, that the Icelandic population is relatively homogenous as regards genetic makeup and environmental parameters.

Our implementation of the Granger causality test is a special form of DBN where there is no causal structure within a single time slice. There are also many variations of the Granger causality test including stationary or non-stationary, dynamic or time-invariant Granger causality tests. Our simple implementation of Granger causality test identified the transcription factor *PER1* as the main causal regulator in the fed time series.

The intra-slice network (SBN) was reconstructed from an independent data set and is fixed in our current model of DBN. Even though the SBN was reconstructed using about 455 samples, there are still many uncertainties about the network structure and edge directions. Further researches on using the SBN as flexible priors for intra-slice structure rather than fixed one are warranted.

Several simulation studies have been carried out to estimate the number of samples that are required to build SBNs or DBNs. Zhu *et al.* [12] showed that these numbers are related to the interaction strength between nodes. For instance, with networks consisting mainly of interactions at intermediate strength, over 80% of interactions in SBN can be recovered at 90% precision with 1000 samples. Similarly, Yu *et al.* [38] showed that over 85% of links in DBNs can be recovered with 2000 samples. In addition, Yu *et al.* showed that the sampling interval is also an important parameter. When the sampling interval is small, the difference between data at consecutive time points will be small. In other words, the independent information added is small. Our time series simulation result (Supplementary Figure 2) and the results of Yu *et al.*, both show that network reconstruction accuracies drop when sampling intervals are large. In both our and Yu *et al.*'s time series simulations, all interactions have the same time lag. In reality, the time lags are different for different transcriptional regulations [39]. Zou and Conzen [3] showed that a better reconstruction accuracy of DBN could be achieved when considering time lag differences. The general DBN model shown in Figure 1a can represent mixed time lags with intra-slice interactions for zero or short time lags and inter-slice interactions for large time lags. Based on the complications discussed above, at least 1000 data points are needed to reconstruct an adequate DBN. Sachs *et al.* [40] suggests that even over 23,000 data points are not sufficient for reconstructing an accurate DBN. Obviously, additional priors can improve reconstruction accuracies with the same amount of data [3],[12]. To accurately estimate the amount of data required to reconstruct DBNs under different interaction strengths using different mixtures of time lags and different priors, a systematic data simulation is warranted.

The causal networks derived from either the Granger causality test or the Dynamic Bayesian network, both showed that the networks under the fasting state were fragmented (loosely connected) while the networks in the feeding state are more highly interconnected. It is well established, that the circadian rhythm interacts with metabolic [32] and immune response processes in rodents [41]. For instance TNF-alpha, which regulates immune cells and induces apoptotic cell death, is also shown to regulate key genes in the circadian rhythm, including Dbp and Per1-3 [41]. It is possible that increasing nutritional load directly affects the circadian rhythm system, possibly through ghrelin [42]. Our results in humans are consistent with the rodent data, showing that feeding is directly linked to the circadian rhythm system. Furthermore, our results suggest that the interconnections between different biological processes such as metabolic and immune responses and activated cell death are weak in the fasted state, while feeding dramatically enhances the interconnections between these different biological processes. Further experimental work is warranted to verify whether these changes still hold in the general population.

Human peripheral blood is the most readily accessible human tissue for clinical studies. Our work on peripheral blood has demonstrated that feeding or increasing nutritional load affects the human circadian rhythm system, which becomes highly connected to other biological processes including metabolic and immune responses. And these effects can be observed in peripheral blood. We believe the results of the present work have broader implications for studies of drug response and for genetic and experimental studies on blood chemistry and vascular related clinical traits. Our results suggest that how blood networks respond to feeding is an important variable that may bring us closer to dissecting the underlying causes of obesity and associated disorders. Our results also provide a guideline on how much data are required for inferring causal relationship in human blood for future experiments.

## Materials and Methods

### Time series data

40 healthy participants from an Icelandic company were recruited to participate in a randomized, two-arm, cross-over study to examine the effects of fasting and feeding on human blood gene expression [18], shown in Figure 2. For the first period of the study the 40 participants were randomized to two treatment groups, with 20 individuals making up each group. All participants began fasting at 9pm the night before the first period of the study. The first treatment group comprised the fasted arm of the study for the first period, where participants continued to fast through the day for the duration of the study (participants were only allowed to drink water during this time). The second treatment group comprised the fed arm of the study for the first period, where participants were fed a standard meal in the morning and then fasted through the rest of the day for the duration of the study. The second period of the study was carried out one week later from the start of the first period. The protocol for the second period of the study was identical to the first period, except those in the fasted arm for the first period were switched to the fed arm, and those in the fed arm for the first period were switched to the fasted arm. Figure 2 shows the schematic for the experimental design.

A total of 560 peripheral blood samples were collected from the 40 participants at 7 time points for each period of the study. Significant inter-individual variation has been noted in human blood gene expression profiles [43]. Previous analyses carried out on this data set detailed the inter-individual variation and overall expression differences between the fasted and fed conditions [18]. In the present study we focus mainly on using temporal information to infer causal relationship by applying a Granger causality test and a dynamic Bayesian network so that possible causal drivers of dynamic changes can be identified from the causal networks. To correct for the individual differences in gene expression we referenced each individual expression profile to the corresponding individual profile at time point 0. This reduced the effective number of time points to 6 for this study.

### Constructing causal networks using Granger's causality test

The time series based causality test was proposed by Wiener [44] as the notion that, if the prediction of one time series could be improved by incorporating the knowledge if a second one, then the second series has a causal influence on the first. Granger was the first to formalize the idea in the context of linear regression model [17], so that time series based causality test is generally referred as Granger causality test. There is a variety of models for testing Granger causality, such as multivariate autoregressive model (MVAR) and bivariate autoregressive model (BVAR). If coefficients in the regression model do not change depending on time, the model is referred as a stationary model. Otherwise it is referred a non-stationary model. The simplest model is stationary bivector autoregressive model. Even though comparing to MVAR, BVAR tends to infer many indirected links, the causal directions of these inferred links follow causal information flows [45]. To remove potential in-direct links, for each gene, we only keep one causal link pointing to it, which has the most significant p-value in the BVAR model.

Traditionally, Granger causality test is applied to long time series. However, it is hard to collect long time course data from human samples. Our data consists of many short time series from multiple individuals. There are several theoretical studies related to combining multiple time series in a general regression frame work, including for instance that of Berhane & Thomas [37] and Guerrero & Pena [46]. Berhane & Thomas [37] proposed to use a mixed-effects model to combine time series from different locations, while Guerrero & Pena [46] outlined a weighted least squares approach. In both approaches, some constraints were applied after a number of assumptions were made.

Our approach is a simplified version of the Berhane & Thomas approach [37]. Instead of using community-specific slopes, we assumed response slopes for individuals are similar. Further, in order to reduce individual specific variation which could affect the response slope, an individual's gene expression data were normalized according to its own expression data at the first time point. Our simulation study shows that causal relationship can be accurately inferred by combining these short time series.

### Simulation of short time series

Under first order stationary BVAR model, a set of data was simulated for causal relationship 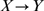 as following:
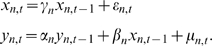
(1)There are 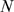 independent time series of length 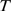, 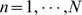, 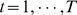. All coefficients and noises follow normal distributions as
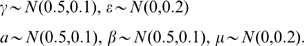
(2)The initial conditions are draw from an uniform distribution with mean 0. 1000 independent time series were simulated, and each series consists of 240 time point (shown as Supplementary Figure 1).

The test of Granger causality 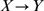 under BVAR model can be carried out by comparing the full model
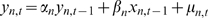
(3)with the autoregressive model
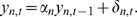
(4)The significance of the Granger causality test 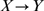 (full model explains more variance than the autoregressive model) is then measured by F-test statistics
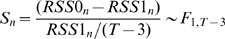
(5)where 
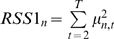
 and 
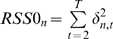
 are sum of squared residuals of full model and autoregressive model, respectively; and 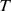 is the length of the time series.

For the 1000 time series simulated above, the p-values of Granger causality 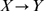 are estimated as Eq. 5. If only partial time points are used, then the power to detect Granger causality decreases (shown in Supplementary Figure 2). It is worth to note when the same number of time points are used, it is more likely to inferred correct causality if the interval between time points is shorter.

If only 6 time points are used, no Granger causality test is significant if considering the time series independently. If assuming 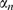 and 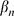 are similar, then these short series can be combined together to infer Granger causality, and the Eq. 3 can be modified as
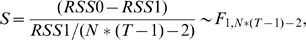
(6)where 
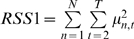
 and 
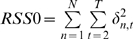
 are sum of squared residuals of full model and autoregression model, respectively. For example, a virtual time series by combining the first 6 time points of randomly selected 40 time series is as informative as a long time series with the same time points.

To estimate the false positive rate, we permuted the assignment of 1000 time series generated above (for example 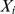 was assigned as 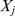 where 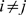) so that the autoregressive assumption was valid. For each permutated data set, we followed the same procedure mentioned above to calculate p-values for the Granger causality test. At different p-value cutoffs, we calculated the recall (positive rate) and the false positive rate (shown in the Supplementary Figure 2).

It is of note that choosing the optimal time lag length in the autoregressive (AR) model normally requires comparing model residuals and statistics at different p-value thresholds. However, because of the small sample size (40) and limited number of time points (6), we restricted our analyses here using AR models with only first order time dependency, similar to what has been done in previous studies [5]–[6]. Similarly, we assumed the Granger causal relations were stationary from time point 1 to 6. That is, we were mainly interested in the mean α and β values in Eq.(1), which represent the averaged Granger causality between genes from time point 1 to 6.

### Bootstrapping test

A bootstrapping procedure of re-sampling individuals with replacement, was used. At each time, one subject (along the associated data at 6 time points) was sampled from a pool of 40 individuals. A bootstrapped data set consisted of 40 sampled individuals (40×6 data points). The same Granger causality test outlined above was applied to the re-sampled data. The bootstrapping procedure was performed 100 times. The link confident value is the percentage of a link's p-values above a multiple testing corrected threshold in the results of the 100 bootstrapping tests.

### Reconstructing the static Bayesian network

455 male samples in IFB cohort [10] was used in reconstruction of the static Bayesian network. A set of informative genes were identified as follows: (1) a gene expressed in the blood (with mean log intensity >−1.5), (2) the variation of the mean log ratio was larger than 1.23. Of the 23720 genes represented on the microarray, 7310 were selected for inclusion in the network reconstruction process as previously described [12],[35]. One thousand Bayesian networks were reconstructed using different random seeds to start the reconstruction process. From the resulting set of 1000 networks generated by this process, edges that appeared in greater than 30% of the networks were used to define a consensus network.

### Reconstructing dynamic Bayesian networks

For a two-slice dynamic Bayesian network represented in Figure 1A, it can be decomposed as 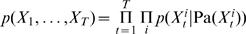, where 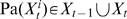 is the parent set of 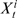. The static Bayesian network reconstructed above was used as the intra-slice network. The intra-slice network is fixed and is not refined in the process of reconstructing dynamic Bayesian networks. Thus, only inter-slice links (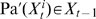) are added or removed during the reconstruction process. Similar to the static Bayesian network reconstruction process, 1000 networks were reconstructed using different seeds and the Bayesian information criterion (BIC) score [47] was used for the optimization. Edges appeared in 30% of the 1000 structures are included in the final network.

## Supporting Information

Figure S1 A Montage display of independently simulated time series for *X*→*Y* based on Equation 1. Each time series consists of 240 time points (only the first 50 points are shown here). Blue lines are for *X*, and red lines are for *Y*.
(0.06 MB EPS)

Figure S2 Prediction accuracies of Granger causality *X*→*Y* using the simulated time series shown in Figure S1. Each full series consists of 240 time points and each short series consists of 6 time points.
(0.03 MB TIF)

Figure S3 The distributions of bootstrapping confident values of links inferred in both fast and fed Granger causality networks. (A) 80% links in the fast network have confident values above 0.5 (B) 90% of links in the fed network have confident values above 0.5.
(0.12 MB TIF)

Figure S4 The out-degree distributions of both fasted and fed Granger causality networks exhibit scale-free properties. (A) The out-degree distribution for the fasted network; (B) the out-degree distribution for the fed network.
(0.03 MB PDF)

Table S1 Inferred causal links in the fast blood Granger causal network.
(0.03 MB TXT)

Table S2 Inferred causal links in the fed blood Granger causal network.
(0.01 MB TXT)

Table S3 Inferred inter-slice causal links in the fast blood Dynamic Bayesian network.
(0.02 MB TXT)

Table S4 Inferred inter-slice causal links in the fed blood Dynamic Bayesian network.
(0.02 MB TXT)
