# Supplementary figures and images for: Characterizing Dynamic Changes in the Human Blood Transcriptional Network
Source: PLoS Comput Biol. 2010 Feb 12;6(2):e1000671. doi: 10.1371/journal.pcbi.1000671 (PMC2820517; doi:10.1371/journal.pcbi.1000671)

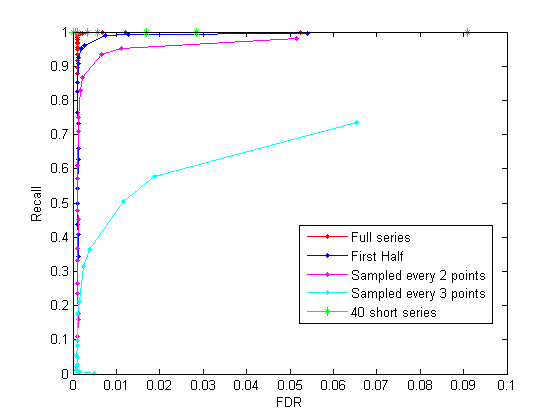

Supplement: Figure S2 — Prediction accuracies of Granger causality X→Y using the simulated time series shown in Figure S1. Each full series consists of 240 time points and each short series consists of 6 time points. (0.03 MB TIF) [file pcbi.1000671.s002.tif]

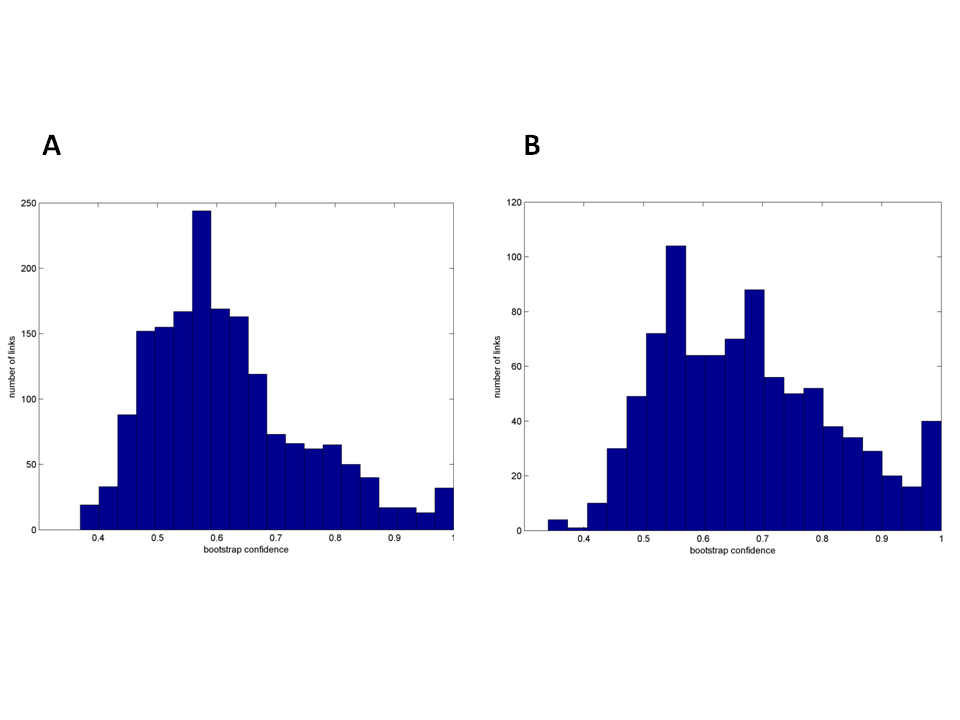

Supplement: Figure S3 — The distributions of bootstrapping confident values of links inferred in both fast and fed Granger causality networks. (A) 80% links in the fast network have confident values above 0.5 (B) 90% of links in the fed network have confident values above 0.5. (0.12 MB TIF) [file pcbi.1000671.s003.tif]

**A**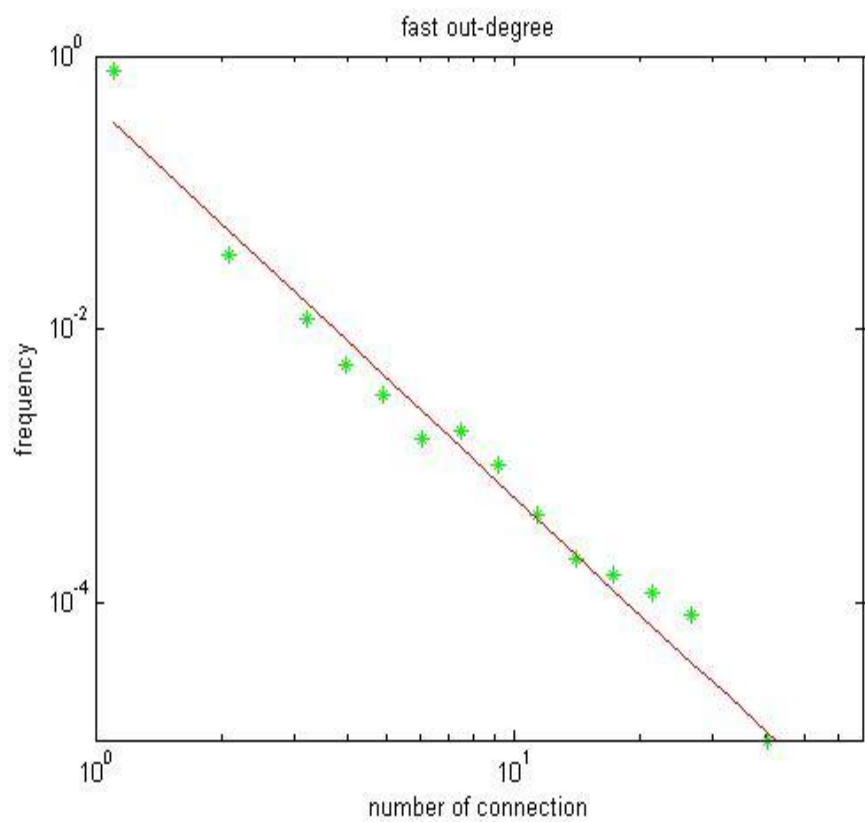**B**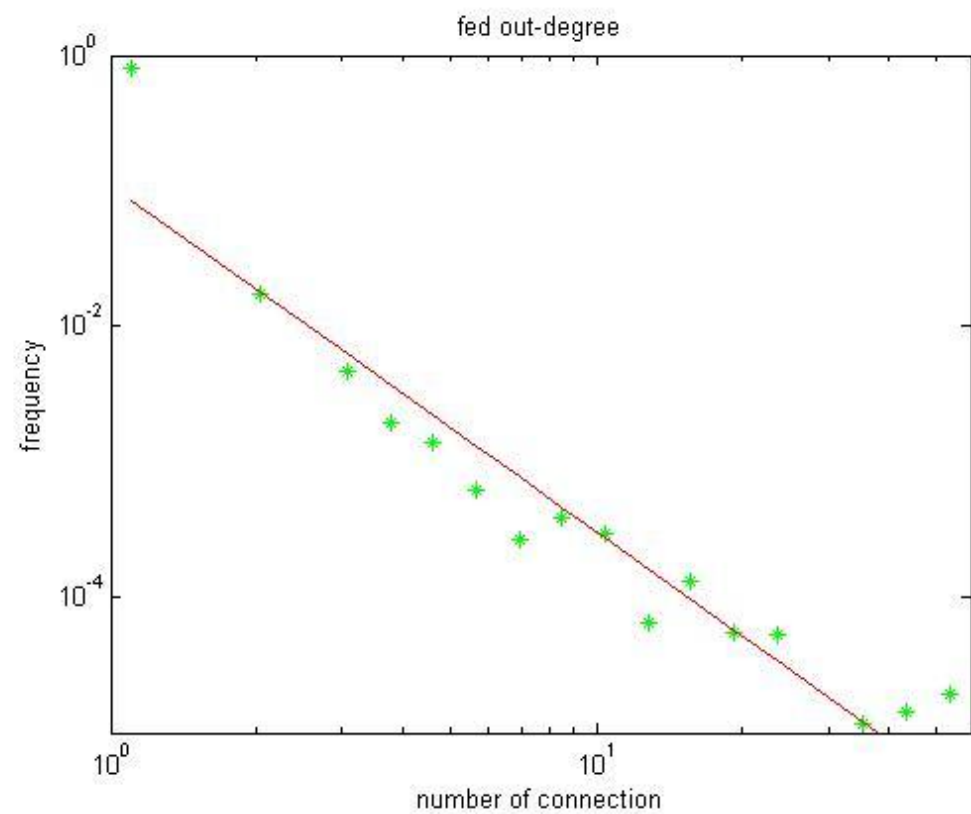

Supplement: Figure S4 — The out-degree distributions of both fasted and fed Granger causality networks exhibit scale-free properties. (A) The out-degree distribution for the fasted network; (B) the out-degree distribution for the fed network. (0.03 MB PDF) [file pcbi.1000671.s004.pdf]
